# Recapitulating Cardiac Structure and Function In Vitro from Simple to Complex Engineering

**DOI:** 10.3390/mi12040386

**Published:** 2021-04-01

**Authors:** Ana Rita M. P. Santos, Yongjun Jang, Inwoo Son, Jongseong Kim, Yongdoo Park

**Affiliations:** BK21 Graduate Program, Department of Biomedical Sciences, College of Medicine, Korea University, Seoul 02841, Korea; Anasantos@korea.ac.kr (A.R.M.P.S.); jyj727@korea.ac.kr (Y.J.); inuinku@korea.ac.kr (I.S.)

**Keywords:** cardiac tissue engineering, human pluripotent stem cell, cardiomyocyte, maturation, extracellular matrix, geometry, computational modeling

## Abstract

Cardiac tissue engineering aims to generate in vivo-like functional tissue for the study of cardiac development, homeostasis, and regeneration. Since the heart is composed of various types of cells and extracellular matrix with a specific microenvironment, the fabrication of cardiac tissue in vitro requires integrating technologies of cardiac cells, biomaterials, fabrication, and computational modeling to model the complexity of heart tissue. Here, we review the recent progress of engineering techniques from simple to complex for fabricating matured cardiac tissue in vitro. Advancements in cardiomyocytes, extracellular matrix, geometry, and computational modeling will be discussed based on a technology perspective and their use for preparation of functional cardiac tissue. Since the heart is a very complex system at multiscale levels, an understanding of each technique and their interactions would be highly beneficial to the development of a fully functional heart in cardiac tissue engineering.

## 1. Introduction

Cardiac tissue engineering is the field of fabricating tissues that mimic the structural and functional characteristics of the heart to study cardiac development, homeostasis, and regeneration. The heart is composed of various types of cells and extracellular matrices in a 3D structure, which hints at the importance of structural integrity and microenvironment [1,2]. Various technologies, such as gene editing, cell assembly, 3D bioprinting, and computational modeling, have been employed to model such complexity [3]. The development of recent technologies integrating cardiac cells, biomaterials, and fabrication will help to advance cardiac tissue engineering [4,5]. Thus, it will be highly beneficial to understand the pros and cons of each technology, aiming at developing fully mature cardiac tissue. In this review, technologies for rebuilding cardiac tissue in vitro will be discussed, from simple to more complex.

Cardiomyocytes, comprising heart muscle, play a central role in heart function and are specialized by long chains of sarcomeres, which are contractile units of the muscle cell [6]. In addition, a high mitochondrial density of cardiomyocytes allows for quick generation of adenosine triphosphate (ATP), which empowers them to function consistently throughout life [7]. From a developmental perspective, cardiomyocytes are terminally differentiated cells with limited proliferative potential, which hampers the progress of cardiac tissue engineering [8]. Therefore, the technology to produce a sufficient number of cardiomyocytes was developed in conjunction with cell reprogramming. The use of induced pluripotent stem cells (iPSCs) for the preparation of cardiomyocytes is an example of such efforts, enabling the unlimited supply of cardiomyocytes for the formation of cardiac tissue [9]. Despite the remarkable success, improving the maturation of cardiomyocytes in morphology, electrophysiology, and metabolism is another hurdle to establishing intact cardiac tissue [10]. The maturation of cardiomyocytes was demonstrated by the application of other components, geometrical guidance, and force.

The cardiac extracellular matrix (ECM) is a three-dimensional network of structural and nonstructural proteins where cardiomyocytes reside with other types of cardiac cells [2]. In addition to its role as a physical scaffold, cardiac ECM plays an important role in the transduction of biochemical and biophysical signals between cardiac cells, resulting in the maturation and homeostatic function of cardiomyocytes [11,12]. This suggests that the integration of a suitable ECM system into cardiac tissue engineering is a prerequisite for the preparation of mature cardiac tissue. Although different types of biomaterials, from naturally derived to synthetic ones, have been utilized to mimic in vivo cardiac microenvironment, there are unfulfilled commitments to date. The use of synthetic composites or decellularized ECMs is an attempt to attain the desired ECM materials by providing enhanced structural support and biological signals [13]. The advancement of ECM materials allows for better cardiac tissue assembly and maturation, and geometrical guidance and force present in the microstructure of the native myocardium are also required.

Cardiomyocytes are anisotropically aligned with cardiac fibroblasts in the myocardium, forming cardiac muscle fibers with gab junctions [14]. In particular, each cardiomyocyte is joined at the end by intercalated disks to construct long fibers supported by fibroblasts that regulate cardiac ECM and electrical currents in a cardiac conduction system [15,16]. Therefore, the geometrical alignment (or guidance) of cardiac cells is an important factor in the construction of mature cardiac tissue. It is known that geometrical guidance induces anisotropic alignment of cells and leads to the structural and functional maturation of cardiac tissue [17,18]. The application of physical force to cardiac tissue is another type of geometrical stimulation since cardiomyocytes deliver tensional force to each other [19]. A microphysiological system, such as an organ-on-a-chip, is a useful platform to investigate the effect of geometrical cues and physical force on cardiac tissue.

Despite continuous advancements in cardiac tissue engineering, most experimental outcomes are still far from mirroring the workings of a real heart. The complexity of human physiology, including hemodynamics and multiscale interactions (from proteins to organs), could be one of the reasons. Since computational simulation allows for many quick and easy virtual experiments, computational modeling of a heart from multiple perspectives such as calcium current, cell‒cell interaction, and blood circulation were developed [20,21,22]. Furthermore, electrophysiology and the mechanical movements of cardiac tissue were demonstrated to understand the physiology of the heart.

**Figure 1:**
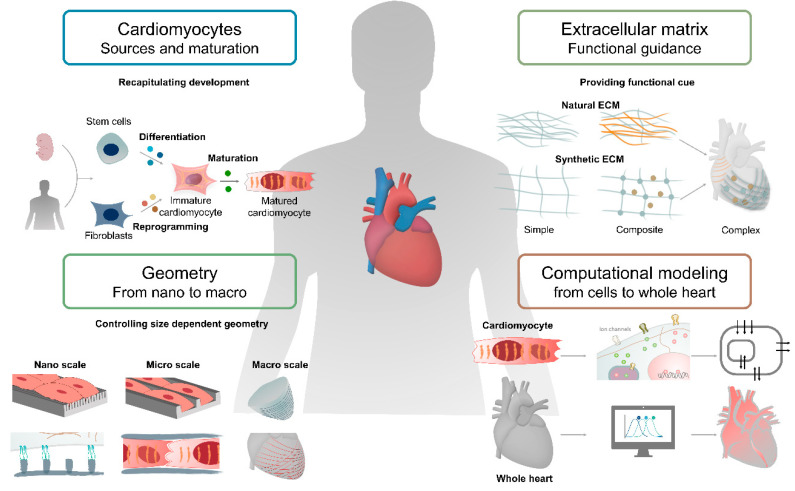
Strategies to model heart function and structure in cardiac tissue engineering.

In this review, we introduce key techniques for the fabrication of cardiac tissue, focusing on components used to model the heart, such as cardiomyocyte, cardiac ECM, and geometry. Next, we discuss how computational modeling of the heart provides a novel perspective to better understand the physiology of the heart, which allows for better design rules for the preparation of matured cardiac tissue. We also describe how to recapitulate the function and structure of the heart by utilizing the recently developed techniques and applications for advances in the field of cardiac tissue engineering (Figure 1).

## 2. Cardiomyocytes

Cardiac tissue engineering aims to generate three-dimensional tissue with high fidelity for the study of heart function and the replacement of damaged cardiac tissue. Cardiomyocytes, one of the major cells in the heart, play a pivotal role in heart function and their loss can lead to many cardiovascular diseases. For this reason, it is essential to study cardiomyocytes for the advancement of cardiac tissue engineering. However, adult mammalian cardiomyocytes are terminally differentiated cells that have a limited regenerative capacity and cannot be obtained in sufficient numbers for studies, so researchers focused on obtaining stable cell sources. In this section, types of cell sources and technologies for establishing matured cardiomyocytes will be reviewed. Various types of cells are used for cardiac tissue engineering from not only primary cells, such as neonatal rat ventricular myocytes (NRVM) and porcine myocytes, but also differentiated cells including pluripotent stem cell (iPSC)-derived cardiomyocytes (Table 1).

In addition to the two-dimensional (2D) differentiation of cardiomyocytes, using human pluripotent stem cells (hPSCs), three-dimensional (3D) differentiation techniques were also developed. Furthermore, patient-derived cells or mutant cells created by gene editing techniques are also used in cardiac disease modeling.

### 2.1. Establishment of Cardiomyocyte Differentiation Using Rodent Cells

#### 2.1.1. Primary Rodent Cardiomyocytes

Cardiomyocytes isolated from animal models have long been used in in vitro studies to understand cardiac physiology and disease. Primary adult ventricular myocytes were isolated from small rodents using the Langendorff method, in which an enzyme solution was injected into the aorta through retrograde perfusion [23], and were used to study contractility-related factors, such as β-adrenergic receptors [24,25], nitric oxide [26,27,28], and ion channels [29,30]. However, neonatal rat ventricular myocytes (NRVMs), when compared to adult rat ventricular myocytes (ARVMs), had the advantage of being isolated in high yield and purity by the enzymatic dissociation method [23]. Furthermore, since NRVMs are immature compared to ARVMs, these cells are actively used as a major cell source for studies including cardiac maturation [31,32,33], development [34,35,36], disease modeling [37,38], and drug screening [39,40].

#### 2.1.2. Mouse Embryonic Stem Cell-Derived Cardiomyocytes

**Figure 2:**
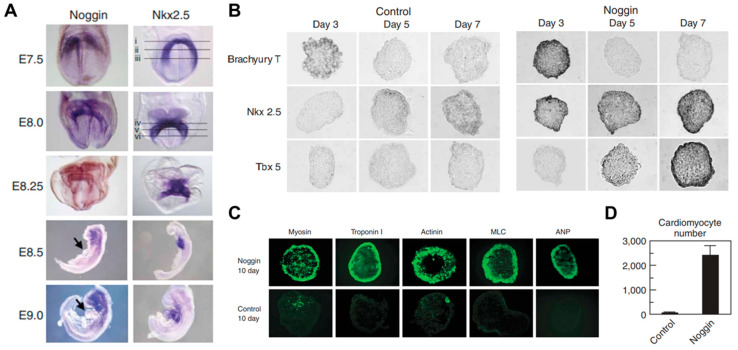
(**A**) Transient expression of noggin according to mouse embryo stages in whole in situ hybridization of noggin and Nkx2.5. (**B**) Section images of embryoid bodies in the control group and noggin-treated group. (**C**) Immunostaining of embryoid body with cardiomyocyte-specific antibodies. (**D**) The number of cardiomyocytes in the embryoid body. Reprinted with permission from Ref. [47]. Copyright 2005 Nature Publishing Group.

Embryonic stem cells (ESCs) provide a virtually unlimited variety of cells for research, enabling an understanding of the development process by overcoming the limitations of an insufficient number of cells. Mouse embryonic stem cells have been used for decades to study cardiogenesis. Wobus et al. generated spontaneously beating mouse cardiomyocytes from embryonic stem cells (mESC) through embryonic bodies (EBs) made by the aggregation of stem cells [41]. The cardiac differentiation through EB formation appeared to be a result of cell‒cell interactions and signal pathways through adrenoceptors, cholinoceptors, and L-type Ca^2+^ channels. However, spontaneously differentiated cardiomyocytes, via the embryonic body, led to heterogeneous cell types such as pacemaker-, atrium-, and ventricle-like type [42]. Wobus et al. showed that retinoic acid increases the mESC-derived cardiac differentiation rate and enhanced the induction of ventricular cardiomyocytes [43]. In addition, Zandstra et al. developed a scalable differentiation system of cardiomyocytes using genetically engineered mESCs. Transfected mESCs with an α-cardiac myosin heavy chain promoter, driving the aminoglycoside phosphotransferase (neomycin resistance) gene, were used for the selection of differentiated cardiomyocytes. Furthermore, understanding the regulatory mechanisms of heart development in vivo is necessary to recapitulate heart development and control the differentiation of stem cells. Several key signals such as bone morphogenetic protein (BMP), Wnt, and FGF, involved in heart development, were studied in mammals [44,45,46]. In particular, Yuasa et al. found that the BMP antagonist noggin was expressed in the heart during development, but it was transient (Figure 2). Transient inhibition of BMP signaling by noggin had a strong effect on cardiomyocyte development and suggested that suppressing and promoting biological signals at specific timings is crucial for cardiomyocyte differentiation [47].

### 2.2. Generation of Cardiomyocytes from Human PSCs

Cardiac research has been advanced based on rodent cardiomyocytes; however, there are limitations because of the differences between rodents and humans [48]. To address these limitations, advanced stem cell differentiation techniques have been applied to generate a reliable source of human cardiomyocytes [49]. The development of human embryonic stem cells (hESCs) and the discovery of induced pluripotent stem cells (iPSCs) enable the supply of human-derived cardiomyocytes, which had a significant impact on advances in the field of cardiac tissue engineering [50].

#### 2.2.1. Establishment of Human PSC-Derived Cardiomyocyte

**Figure 3:**
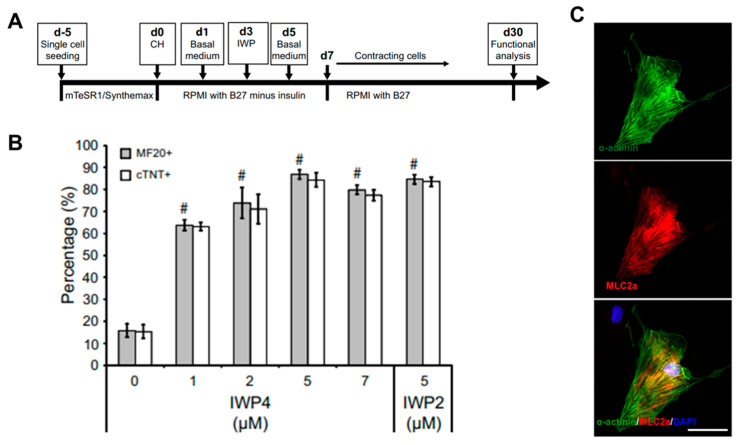
(**A**) Schematic protocol for differentiation of human pluripotent stem cells (hPSCs) into cardiomyocytes. (**B**) Differentiation of cardiomyocytes as a function of IWP4 and IWP2. (**C**) Immunostaining images of cardiomyocyte differentiated from hPSCs (scale bar: 50 μm). ^#^ *p* < 0.005, each point versus no drug; Student’s *t* test. Reprinted with permission from Ref. [53]. Copyright 2021 National Academy of Sciences.

The most reproducible and effective differentiation method using hPSCs is to recapitulate important steps during cardiac development in early embryos [51]. Mummery et al. generated high-yield and highly purified human cardiomyocytes by using activin A and bone morphogenetic protein 4 (BMP4) to induce cardiac mesoderm of hESCs [52]. Through this method, cardiomyocytes with high purity of about 82.6% were generated, and these cells were transplanted into the heart of an infarcted rat model to show that the lesions of the heart were partially remuscularized. In addition, Kattman et al. generated enriched cardiomyocytes in mice (>60%) and hiPSCs (>50%) by controlling Activin/Nodal and BMP4 signaling pathways along with monitoring the cardiac mesoderm markers Flk-1/KDR and PDGFR-α. Through stage-specific optimization studies based on the concentration of activin A and BMP4, the optimized protocol showed that the main developmental steps of PSC-derived cardiac differentiation can be quantified and standardized. However, the protocol using activin A and BMP4 to induce cardiomyocytes should be optimized individually for each cell line and the differentiation efficiency can be highly variable depending on cell lines. To solve these problems, Lian et al. recapitulated the biphasic effect of Wnt signaling on cardiac development in mice and zebrafish and on the cardiomyocyte differentiation of hPSCs and showed that temporal modulation of Wnt signaling induced cardiomyocyte differentiation in hPSCs with extremely high yields of up to 98% [53]. Furthermore, since the robust differentiated cardiomyocytes were generated under defined, growth factor-free conditions with small molecules (CHIR99021 and IWP2) instead of growth factors, they could lead to a reduction of costs and increased reproducibility in generating cardiomyocytes from hPSCs (Figure 3). Burridge et al. developed a chemically defined cardiac differentiation platform from hiPSCs [54]. To generate hiPSC-derived cardiomyocytes under chemically defined conditions, a synthesized vitronectin peptide substrate was used as a surface coating material rather than matrigel and CDM3 (a chemically defined medium), made by adding recombinant human albumin (rHA) and L-ascorbic acid 2-phosphate to the existing RPMI medium. The cardiac induction molecules were CHIR99021, which activates Wnt signals, and Wnt-c59, which inactivates Wnt signals; this protocol was tested in 11 hiPSC lines with an up to 95% cardiomyocyte yield. These studies suggested that a protocol in a chemically defined condition, with small molecules to induce cardiomyocyte differentiation from hPSCs, was established, although the differentiated cells remained immature.

#### 2.2.2. The Major Properties of Matured Cardiomyocyte

The widespread engagement of hPSC-derived cardiomyocytes (CM) differentiation techniques led to advances in the field of cardiac tissue engineering. However, the major obstacle in their application is that these are immature cardiomyocytes [10]. During mammalian heart development, cardiac cells undergo a highly dynamic process, including specification, morphogenesis, and maturation. Through this process, mature cardiomyocytes significantly differ from immature cardiomyocytes in terms of their morphological structure, e.g., cell size, junction, and myofibril formation, and biological functions such as electrophysiology and metabolism [55].

**Figure 4:**
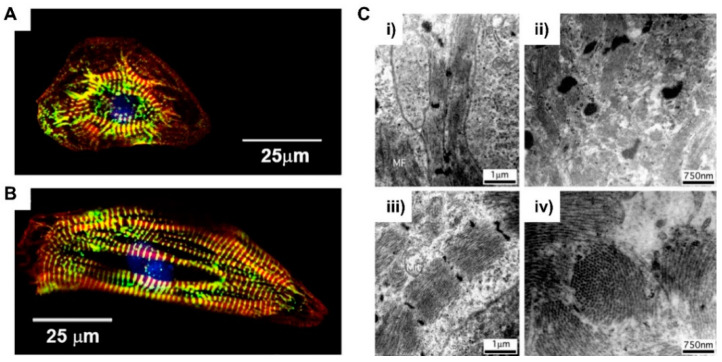
Representative immunofluorescent images of immature cardiomyocyte (**A**) and mature cardiomyocyte (**B**). The cells are stained with a-actinin (green), phalloidin (F-actin, red), and Hoechst 33342 (nuclei, blue). (**C**) Ultrastructure of immature cardiomyocyte sarcomere (**i**) and Z-band (**ii**), and mature cardiomyocyte sarcomere (**iii**) and z-band (**iv**). Reprinted with permission from Refs. [56,59]. Copyright 2019 Elsevier B.V. (Amsterdam, Netherlands) and Copyright 2003 the American Physiological Society, respectively.

When it comes to their morphology, hPSC-derived cardiomyocytes have a round shape that is morphologically similar to early fetal cardiomyocytes (Figure 4A) [56]. Unlike the fetal period, which was grown through the mitotic cell cycle, postnatal cardiomyocytes undergo hypertrophic growth and increase in size by about 30- to 40-fold [57]. The volume of adult cardiomyocytes is about 40,000 μm^3^, while immature cardiomyocytes are 2000 μm^3^ [58,59]. This hypertrophic growth of cardiomyocytes is closely related to the development of their morphology. The cardiomyocyte is anisotropically elongated and polarized in shape, like adult cardiomyocytes (Figure 4B) [56]. In addition, cardiomyocytes begin to form junctions with adjacent cells and ECMs, characterized by intercalated discs containing gap junctions that induce cell‒cell electrical coupling [60]. Connexin 43, one of the major gap junctions in cardiomyocytes, is expressed and localized in the intercalated discs of adult cardiomyocytes, which promotes electrical conduction and contraction compared to nonpolarized cardiomyocytes [61,62]. Myofibril formation is essential for development of contractile forces in cardiomyocytes. Sarcomeres, the longitudinally repeated contractile unit of myofibrils, are more organized in adult cardiomyocytes when compared to PSC-CMs (Figure 4C) [59,63]. In adult cardiomyocytes, sarcomeres are highly dense and aligned with a spacing of about 2.2 μm [64]. Sarcomeres are composed of thin filaments, thick filaments, and associated proteins, including titin. In sarcomere, actinin forms a Z-line and myomesin forms a M-line. This myofibril formation and organization depends on the expression of structural proteins, such as α-actinin, myosin heavy chain, titin, and the troponin complex [65].

Contraction and relaxation of cardiomyocytes is generated by electrical stimulations called cellular action potentials that require tight control of cardiac ion channels [66]. The action potential of cardiomyocytes results from the differences between intracellular and external potentials via sarcolemmal ion channels. The potential of immature cardiomyocytes is −60 mV and that of mature cardiomyocytes is −85 mV, which generates differential functions in terms of electrophysiology [67]. This difference arises from insufficient development of ion channels, such as internal rectifier potassium channel (Kir2.1), voltage gated sodium channel (Nav1.5), and L-type Ca^2+^ channel (Cav1.2). In addition, since membrane depolarization is related to the regulation of intracellular Ca^2+^ concentration, it affects sarcomere contraction. In addition to the calcium ion channels present in the cell membrane, the intracellular Ca^2+^ concentration is regulated by the ryanodine receptor 2 (RYR2) of the sarcoplasmic reticulum (SR), the sarco/endoplasmic reticulum Ca^2+^-ATPase (SERCA2), and the Na^+^-Ca^2+^ exchanger (NCX) [68]. During heart development, cardiomyocytes undergo metabolic shifts in glycolysis to oxidative metabolism, which is accompanied by changes in the morphology of mitochondria [69]. As the amount of circulating blood increases, the level of lipids and oxygen in the blood increases and plays an important role in mediating metabolic reprogramming, constantly increasing the rate of ATP production [70]. Immature cardiac mitochondria are located around the nucleus; they are small in both size and numbers and have a low density of cristae. In contrast to immature mitochondria, mature mitochondria increase significantly in size and number through fusion and division, accounting for about 40% of the cell volume [71]. It also forms a densely organized crista internally, facilitating electron transport for ATP synthesis, and externally aligns along the muscle fibers and sarcolemma to increase ATP efficiency.

### 2.3. Cell-Based Application for Cardiac Tissue Engineering

#### 2.3.1. Cardiomyocyte Maturation by Biochemical Cues

**Figure 5:**
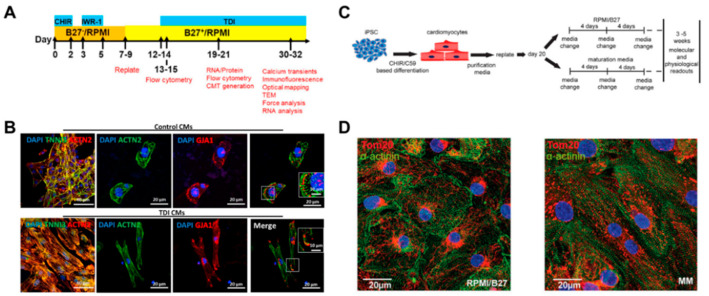
(**A**) Differentiation protocol for cardiomyocytes with TDI (T3, Dex, IGF-1) treatment. (**B**) Immunofluorescence images of cardiomyocytes with or without TDI treatment that show morphological changes and expression of maturation markers. (**C**) Schematic of cardiomyocyte differentiation to metabolic maturation. (**D**) Immunofluorescence images of mitochondria expression by Tomm20 in cardiomyocytes with or without MM (maturation media) treatment. Reprinted with permission from Refs. [73,75]. Copyright 2019 Published by Elsevier Ltd. and Copyright 2020 Elsevier B.V., respectively.

Providing cardiomyocytes derived from human pluripotent stem cells (hPSC-CMs) has led to the ongoing advancement of biomedical research for heart regeneration, cardiotoxicity screening, and disease modeling. The induction of in vivo-like mature hPSC-CM is the first step in their potential application in cardiac regeneration. To promote cardiomyocyte maturation, Parikh et al. applied thyroid hormone T3 (triiodothyronine) and glucocorticoid Dex (dexamethasone) during cardiac differentiation [72]. It was shown that an extensive t-tubule network developed when T3 and Dex were applied together, but not T3 or Dex alone. In addition, the development of the t-tubule-induced structural organization of RYR2 (ryanodine receptor), which increased Ca ion release in cardiomyocytes, resulting in the enhancement of excitation‒contraction coupling of cardiomyocytes. Following the previous study, Huang et al. tested additional biochemical factors, including insulin-like growth factor-1 (IGF-1), to improve the degree of maturation in hPSC-CMs (Figure 5A) [73]. The combination of biochemical factors, such as thyroid hormone, dexamethasone, and insulin-like growth factor-1 (TDI), induced maturation in gene expression profiles and improved the structural and functional characteristics of both 2D and 3D hPSC-CMs (Figure 5B). These studies suggested that treatment with biochemical factors significantly improved the maturation of the structural and functional properties of hPSC-CMs.

Major changes in metabolism as the infant transitions from placental nutrition to nursing also have an impact on cardiomyocyte maturation. Fetal cardiomyocytes produce most of the ATP through glycolysis, but after birth they undergo a metabolic change process that produces ATP, primarily through fatty acid beta oxidation [74]. Yang et al. mimicked this metabolic change and treated fatty acid as an energy source to induce maturation of hPSC-CMs [56]. Fatty acid treatment induced hPSC-CM maturation, especially in cardiac hypertrophy and force generation. Fatty acid treatment also enhanced calcium dynamics, action potential upstroke velocity, and oxidative capacity. In addition, Feyen et al. developed a maturation medium containing physiologically appropriate levels of supplements such as glucose, Ca^2+^, Albumin-bound fatty acid, creatine, L-carnitine, and taurine, to induce metabolic maturation of hPSC-CMs (Figure 5C,D) [75]. The maturation media promoted fatty acid oxidation and affected the electrophysiological and mechanical properties of hPSC-CMs. In addition to increased mRNA expression of KCNH2, KCNQ1, and KCNJ2, known as important potassium channels in cardiac repolarization, genes involved in z-disc singling increased. These studies suggested that the metabolic change of hPSC-CMs via restriction of the energy source can induce maturation of hPSC-CMs.

#### 2.3.2. Chamber-Specific Differentiation by Biochemical Cues

**Figure 6:**
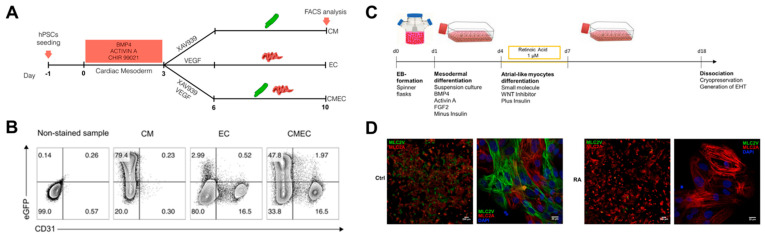
(**A**) Three differentiation protocols to induce cardiomyocytes (CM), endothelial cells (EC), and both (CMEC). (**B**) FACS plots of differentiated CM, EC, and CMEC from NKX2-5^eGFP/w^ hESCs. (**C**) Schematic of cardiomyocyte differentiation with RA (retinoic acid) to induce an atrial-like phenotype. (**D**) Immunofluorescence image of chamber-specific markers (MLC2v, ventricle marker; green and MLC2a, atrial marker; red). Reprinted with permission from Refs. [76,78]. Copyright 2017, 2018 Elsevier B.V.

Advances in differentiation technology have led to the improvement of cardiac differentiation, i.e., the induction of specific cardiomyocyte types and other major cells of the heart. Giacomelli et al. simultaneously differentiated cardiomyocytes and endothelial cells from hPSCs and established the protocol (Figure 6A,B) [76]. They induced initial cardiac mesoderm with BMP4, activin A, and CHIR 99021, and treated XAV939 and VEGF to induce co-differentiation of cardiomyocytes and endothelial cells. In the early cardiac mesoderm state, XAV939 treatment differentiated mesoderm cells into cardiomyocytes, while VEGF treatment differentiated mesoderm cells into endothelial cells. It was also suggested that EC has endothelium identity by expressing cardiac-specific markers such as MEOx2, GATA4, GATA6, and ISL1 in addition to vascular markers such as KDR, VEC, and CD31. In addition, Lee et al. generated atrial-type cardiomyocytes from hPSCs and showed that retinoic acid (RA) signaling is required for atrial specification during the mesoderm development stage [77]. In particular, they found that atrial and ventricular cardiomyocytes could be distinguished based on CD235a and RALDH2 expression in the early cardiac mesoderm. RA signaling induced expression of RALDH2 in the mesoderm, leading to differentiation into atrial-type cardiomyocytes. Furthermore, Lemme et al. generated 3D atrial engineered heart tissue (RA-EHT) based on the retinoic acid protocol (Figure 6C,D) [78]. RA-EHT showed atrial electrophysiological properties such as low force generation and short action potential duration, as well as atrial-specific markers such as COUPTFII and MLC2a in mRNA and protein expression. Based on the specific differentiation protocol of previous studies, Zhao et al. created a 3D chamber-specific cardiac tissue consisting of atrial myocardial cells on one side of the tissue and ventricular myocardial cells on the other side of the tissue [79]. Chamber-specific cardiac tissue showed different gene expression and beating properties according to atrial and ventricle phenotypes, and the response to the drugs was also different. These studies suggested that type-specific cardiomyocyte generation could provide an in vitro model similar to the in vivo one for cardiotoxicity testing, disease modeling, and regenerative medicine.

#### 2.3.3. Gene Editing Technologies for Disease Modeling In Vitro

The development of hPSC-CM differentiation technologies and the convergence of genetic modification technologies made it possible to study genetic disease modeling by modifying specific genes. Familial dilated cardiomyopathy occurs as a problem of sarcomere function, and mutations in the gene encoding for titin (TTN) are known to be one of the main causes. To examine the function of titin in cardiomyocytes, Zaunbrecher et al. generated hiPSCs with mutations in the gene encoding titin (TTN) [80]. In particular, TTN-Z^-/-^-CMs, homozygous mutants of the Z-disc (TTN-Z^-/-^), showed visible contraction and had sarcomere, but TTN-A^-/-^-CMs, homozygous mutants of the A band (TTN-A^-/-^), showed nothing. It was demonstrated that cronos titin, an isoform of titin, is expressed in TTN-Z-CMs and is compatible with partial sarcomere formation and contraction. Since cronos knockout (KO) CM showed disarrayed sarcomere formation with lower contractile force, they suggested that cronos titin is essential for normal myofibril formation development and function. In addition, Brandão et al. generated hiPSC-CMs with mutations in the isogenic set of the KCNH2 gene to study type 2 long QT syndrome (LQT2) [81]. They confirmed that the genetically matched hiPSC lines had different phenotypes, such as electrophysiological characteristics and hERG channel functions, depending on the mutation variation of KCNH2, and this showed that there was a clear difference in the drug-based arrhythmogenesis model. These studies show that in vitro modeling of congenital diseases occurring in the heart is possible through the convergence of genetic modification and hPSC-CM differentiation technologies.

## 3. The Cardiac ECM

The ECM is a complex network of structural and nonstructural proteins. It provides essential cues for diverse cellular responses, including cellular migration, development, and maturation. The ECM of the heart provides a strong elastic anchorage and support for the precise alignment of cardiomyocytes and delivers a specialized cellular environment that permits electrical coupling and cardiac impulse propagation between cells. It also transmits cardiomyocytes’ contractile forces to the surrounding matrix for the continuous blood pumping of the heart. Although the ECM was initially seen as an inert scaffold, its organized and dynamic mesh of proteins is now understood, and while many questions regarding the cardiac ECM are still unanswered, changes in physical properties can influence macroscopic and microscopic cues in cell behavior.

Cardiac tissue engineering is a field that aims to mimic the structure and function of the heart by combining knowledge and techniques from materials science, micro/nanoengineering, cellular biology, and biochemistry. Recently, biomimetic and tissue-specific materials that mimic the native ECM environment by providing appropriate chemical and biological cues were the focus of cardiac tissue engineering. In this section, ECM proteins used for the engineering of cardiac tissue are analyzed from natural to synthetic polymers, considering the complexity of matrix fabrication technologies. Natural materials such as chitosan, collagen, and alginate were used continuously and refined in cardiac tissue engineering due to the advantages of biocompatibility, biodegradability, and nontoxicity. However, these natural materials present certain limitations when it comes to fully functional and structural cellular support. Synthetic polymers can be tailored by changing the composition to provide appropriate biological and chemical cues that resemble the native cardiac tissue from an extracellular matrix viewpoint (Table 2). Here, we will be recapitulating the current usage and evolution of cardiac ECM-focused biomaterials and scaffolds from simple to complex while sharing future perspectives and expectations for cardiac tissue engineering.

### 3.1. Heart ECM Components

#### 3.1.1. Basic Components of the Heart

The extracellular matrix (ECM) of the heart is composed of a variety of proteins including glycoproteins, proteoglycans, and glycosaminoglycans (GAGs) that are classified as shown in Table 3 (adapted from [82]). For instance, the extracellular matrix (ECM) of the heart is primarily composed of collagen, which is one of the most abundant ECM components, and is a key factor in maintaining the structure and integrity of tissue.

There are different types of collagen; overall, they provide the appropriate biological environment for the cells by forming essential mechanical building blocks and providing resistant stretch and tensile stress [83]. Collagen type I makes up about 80% of the total matrix and forms predominant thick rod-like fibers in the perimysium and epimysium [84]. Moreover, collagen type I provides stiffness to the myocardial wall and aids in force transmission for the overall structural framework of cardiac tissue [85]. Collagen type III composes around 10% of the cardiac ECM and, alongside collagen type I, provides the cardiac tissue with high compliance and functional integrity [86]. These collagens take part in cellular signaling and provide structural stability in cardiac tissue. Lastly, collagen V constitutes 5% of the total matrix and is involved in regulating scar size after ischemic injury [87,88].

Moreover, in the cardiac ECM, smaller amounts of laminin, elastin, fibronectin, fibrillin, and collagen type IV are arranged into unique tissue structures, such as endomysial, perimysial, and epimysial layers, conferring the cardiac ECM with biological functions when linked to the surrounding matrix [89,90].

**Table 3:** The major components of the extracellular matrix (ECM) of the heart indexed by structural and nonstructural components. Adapted from [82].

	Structural	Semi-Structural	Non-Structural
**Glycoproteins**	Fiber: Collagens Elastins (not glycosylated) [91]	Fibronectin Laminin [92]	Prototypical matricellular proteins [93]
**Glycosaminoglycans**	Chondroitin sulfate [94]	Hyaluronic acid(Hyaluronan) [95]	
**Proteoglycans**		Basement membrane proteoglycans Cell Surface Proteoglycans Small Leucine Rich Proteoglycans [96,97]	

#### 3.1.2. Cardiac ECM Components in Development and Disease

In early heart development, most collagen fibers and fibronectin are predominantly localized along the epicardial and endocardial layers of the heart [2]. Later in the embryo’s development, due to the increase in fetal growth and higher energy requirements, fibronectin and laminin are expressed and widely distributed in the myocardium [2]. Along with cardiac tissue development, there is a progressive build-up of the ECM, where there is an increase in collagen fiber thickness, and a highly aligned, interconnected, intercoiled, and quickly developed collagen network forms [98,99,100]. Immediately after birth, fibronectin, laminin, and total collagen proportions decrease [98]. In the adult heart’s ECM, collagen types I and III increase; however, the opposite is observed for collagen types IV, V, and VI [101].

**Figure 7:**
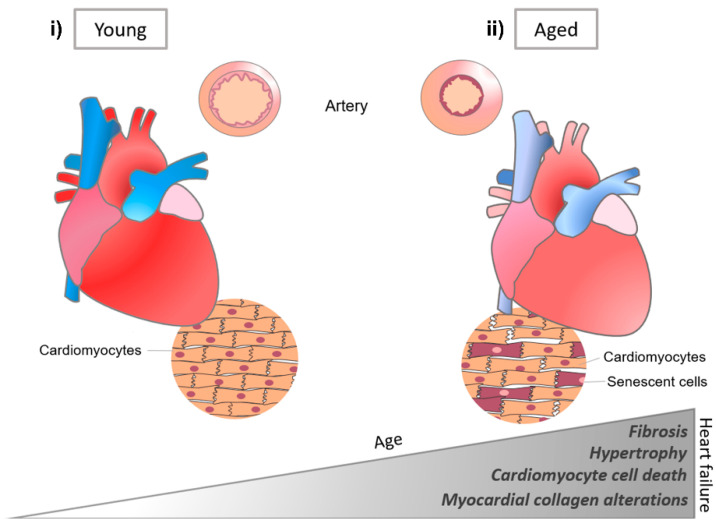
Schematic of the heart by aging. Young and aged hearts show different heart vasculature and tissue integrity. Due to aging, matrix changes in the heart contribute to the development of various cardiovascular diseases leading to heart failure.

Matrix changes in the heart have been related not only to cardiac development, but also to various cardiac diseases (Figure 7) [98]. For instance, collagen ECM plays a key role in cardiovascular processes, and with age, ECM remodeling is closely related to cardiac disease, such as myocardial dysfunction and fibrosis [102]. Remodeling and myocardial failure are generally characterized by collagen accumulation, fibril disruption, impaired rearrangement of the structure, and myocyte loss in the cardiac ECM [86,103]. Accumulation of fibronectin and laminin with aging generates the redistribution of stress in the heart, which affects the mechanical environment, such as diastolic dysfunction [104], reduced systolic performance, and decreased tissue compliance [105,106].

### 3.2. Naturally Derived ECMs in Cardiac Tissue Engineering

The use of the extracellular matrix (ECM) has been adopted by cardiac tissue engineering and regenerative medicine as a bioactive regenerative agent and cellular delivery device. Thus, various ECMs were studied in regenerative medicine and cardiac tissue engineering, where many sources of natural ECMs were assessed for cardiac applications. In this section, we will be exploring novel natural ECM techniques and applications for engineering heart tissue in vitro in disease modeling and drug testing.

#### 3.2.1. Collagen

Collagen is one of the most used natural polymers in cardiac tissue engineering [107]. It has the advantages of biocompatibility and thermal reversibility, and so is often used as a support for cellular activities and widely applied in the study of disease models, such as acute myocardial infarction (MI) [108]. Although novel acute myocardial infarction (MI) therapies are promising, many patients still develop heart failure and adverse cardiac remodeling. Moreover, collagen type I and collagen type III levels are altered according to ischemic heart disease, which induces a pathological fibrosis and changes cardiac function and structure [109]. For this reason, new therapies that can prevent ECM remodeling and support tissue repair are needed. While the majority of studies are in vivo, collagen scaffolds can be utilized in vitro for three-dimensional vascularization promotion [110], cellular regeneration [111], and collagen scaffold composite fabrication [112]. As previously mentioned, collagen scaffolds make good platforms for tissue reconstruction and repair. Studies such as those of Bowlin et al. tested electrospun collagen scaffolds for cellular support and maturation [113,114]. In cardiac tissue engineering and for the repair of impaired hearts, electrospun scaffolds were quite useful in the fabrication of three-dimensional grafts in vitro. In a relevant study, cardiac tissue vascularization and orientation was induced by the development of an electrospun degradable cardiac collagen grafts [115]. More recently, collagen scaffold fabrication was incorporated with other techniques and materials, such as electroactive graphene oxide or alginate microparticles, for the optimization of the physicochemical properties of the scaffold or for the better delivery of chemical cues within the scaffold itself [116,117]. Moreover, next-generation three-dimensional collagen scaffolds that resemble in vivo-like phenotypes of native cardiac tissue [118], which are crosslinked, decellularized [119], porous, and include biochemical factors [120], are being fabricated for the further optimization of collagen scaffolds in cardiac tissue engineering.

**Figure 8:**
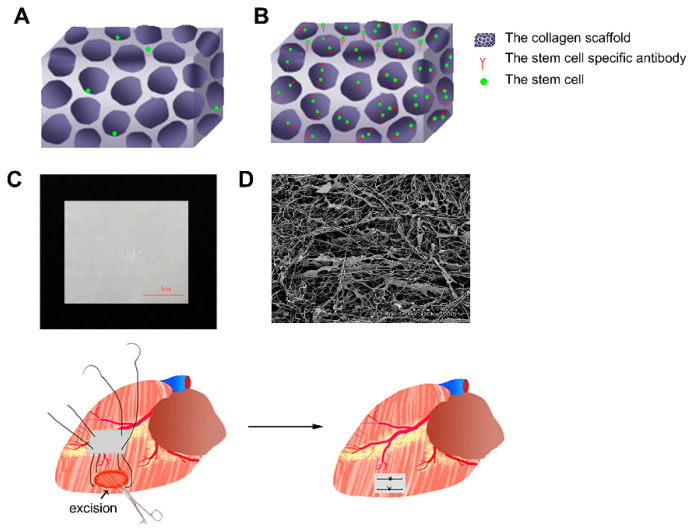
Collagen scaffolds for enhanced tissue regeneration. (**A**) Unmodified stem cells retained on collagen scaffold. (**B**) Addition of stem cell-specific antibody in the collagen scaffold. (**C**) Macroscopic observation of the scaffold. (**D**) SEM image of collagen scaffold. Reprinted with permission from Ref. [122]. Copyright 2010 Elsevier Ltd.

In a study by Mclaughlin et al., injectable recombinant human collagen type I (rHCI) and type III (rHCIII) matrices for treating MI were developed [121]. The results of this study showed cardiomyocyte survival and less pathological remodeling of the myocardium by the promotion of a healing environment, from rHCI, post-MI. The usage of collagen scaffolds for 3D reestablishment and guidance was also studied. Functional collagen scaffolds were created and conjugated with cellular antibodies. These were shown to be beneficial for the promotion of tissue regeneration (Figure 8) [122]. In another study, the repair effect of collagen type I and MaxGel was analyzed upon injection, alone or combined, into Wistar rats with infarcted hearts [123]. Results showed improvement of cardiac function in injected animals, suggesting the capacity to cease the continuous decline of cardiac function in an infarcted heart.

#### 3.2.2. Fibrin

**Figure 9:**
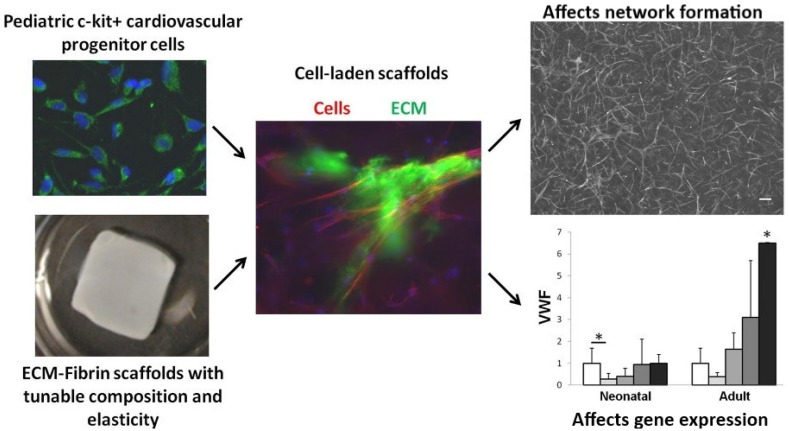
The cardiac ECM‒fibrin hybrid scaffold as an approach to control mechanical and biochemical signals in cell fate. * = Significant difference for *p* < 0.05 (*N* = 3–4 per condition). Reprinted with permission from Ref. [125]. Copyright 2014 Acta Materialia Inc. (Amsterdam, The Netherlands).

Within the components of the cardiac ECM, fibrin, which is formed by a combination of thrombin and fibrinogen, is nontoxic and biodegradable, and thus suited for tissue engineering [124]. The implementation of cardiac ECM within fibrin scaffolds of diversified stiffness was shown to influence cardiac progenitor cell differentiation (Figure 9) [125]. Fibrin-derived ECMs can also be utilized in surgical treatments where there is a risk of tissue adhesion. In a study conducted by Funamoto et al., decellularized pericardium with fibrin glue was used to prevent rat heart adhesion [126]. In this study, a high-hydrostatic pressure method was utilized to decellularize porcine pericardia where cells adhered during an in vitro cell seeding test. However, it was observed that, in the fibrin-coated pericardial ECM, cell adhesion was reduced, proving that fibrin-coated pericardial ECM can be used as an adhesion prevention material for cardiovascular surgery treatment. In an in vivo study, Birla et al. utilized neonatal cardiomyocytes, which were implanted into the femoral artery in adult rats through manufactured fibrin gel tubes [127]. After an implantation period of three weeks, the gel assembled with the fibrin and cardiomyocytes formed a dense capillary network with mature cardiac tissue that possessed regular cardiac functions, such as contractility and synchronous exterior electrical signal pacing. Rat cardiomyocytes were embedded within the fibrin gel and their contractility was maintained for up to two months, showcasing a normal pacing ability [128]. Additionally, the implanted cardiomyocytes’ morphology was shown to be aligned. In a post myocardial infarct, acellular fibrin delivery was shown to improve recovery due to the capability of fibrin to stimulate ECM production in cardiac cells [126]. Furthermore, after myocardial infarction, fibrin patches were shown to improve the function of cardiac cells via induced pluripotent stem cell-derived cardiomyocyte delivery to rats and pigs [129,130].

#### 3.2.3. Matrigel

**Figure 10:**
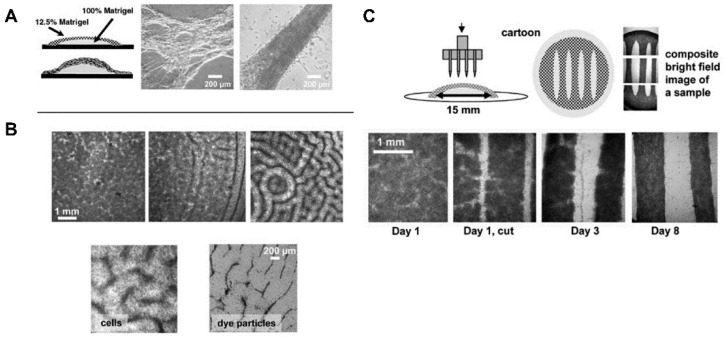
Formation of cardiac fibers in Matrigel. (**A**) Schematic of cardiomyocytes on Matrigel and bright field images of thin and thick fibers formed on the samples. (**B**) Representative images of low, intermediate, and high degree of patterning are observable with different Matrigel concentrations. (**C**) Seeding of cultured cardiomyocytes according to Matrigel pillow protocol. Reprinted with permission from Ref. [134]. Copyright 2008 Future Science Group.

Matrigel is an ECM-mimicking hydrogel produced by mouse Engelbreth‒Holm‒Swarm tumors [131]. It is cytocompatible and resembles the native ECM due to its composition and assembling structure, such as growth factors and basement membrane proteins, including laminin, collagen IV, and enactin [132]. Additionally, compared with other natural hydrogels, Matrigel is capable of faster vascularization [133]. Matrigel is also utilized in in vitro models that are capable of mimicking 3D microenvironments with conditions for mechanical load (Figure 10) [134]. In a study by Zhang et al., a novel modified cardiac explant Matrigel assay was developed. The benefits of this Matrigel were tested under normoxia and hypoxia conditions by observing the ability of the cardiac explants to form vascular sprouts. It was revealed that the morphology of these sprouts was consistent with myocardial capillary formation in vivo [135]. Cardiac progenitor cells (CPCs) derived from embryonic heart tubes were seeded into Matrigel and differentiated into cardiac pacemaker cells after endothelin-1 treatment. Spontaneous beating tissue, namely TECP (transplantation of a tissue-engineered cardiac pacemaker), were obtained [136]. Due to the variation in Matrigel’s matrix composition and the presence of unknown cell signaling factors, studies are often unrepeatable. Furthermore, the medical applications of Matrigel are limited and compromised since it originates from cancerous living tissue.

#### 3.2.4. Chitosan

**Figure 11:**
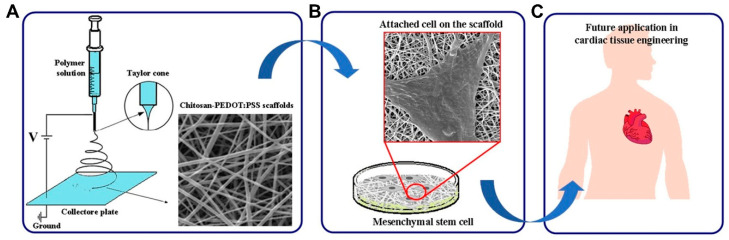
Conductive nanofiber scaffold with Chitosan and PEDOT: PSS. (**A**) Scaffold design and physical property. (**B**) Cell attachment on the scaffold. (**C**) Potential future applications in cardiac tissue engineering. Reprinted with permission from Ref. [143]. Copyright 2019 Elsevier B.V.

Chitosan (CS), a polysaccharide, possesses hydrophilic characteristics and is structurally similar to the glycosaminoglycans in the heart. For these reasons, it garnered much interest in the cardiac tissue engineering field [137]. Chitosan is obtained by chitin’s deacetylation, which is readily available, is low cost, and is the second most abundant polymer in nature [138,139]. Additionally, chitosan is ideal for scaffold manufacturing and development due to its physiochemical properties, such as crystallinity, positive charge, etc. It is also easy to process into varied porous scaffolds and films and is soluble in weak acids of pH < 6.3 [140]. In a study conducted by Shu et al., in order to improve angiogenesis under hypoxia after MI, a chitosan chloride-RoY (CSC1-RoY) hydrogel was developed [141]. The results suggested that RoY peptide’s introduction did not only improve angiogenesis in the MI region but can also enhance overall cardiac function. Chitosan can also be utilized based on fabrication trends, such as the creation of a three-dimensional macroporous cardiac patch from CS and decellularized myocardium ECM [142]. Different concentrations of CS and ECM were analyzed for their effect on mechanical strength, pore size, cell viability, and the biodegradability of the patch, and the results suggested that such a scaffold can transmit chemical and mechanical cues native to the cardiac tissue and support the growth of CPCs, proving its potential in cardiac tissue engineering. Recently, chitosan scaffolds containing other polymers, such as PEDOT:PSS, were studied (Figure 11) and it was found that such a combination enhanced the electrical conductivity and mechanical properties of the electrospun scaffolds, not only improving their biocompatibility but also making them suitable scaffolds for a variety of applications [143].

#### 3.2.5. Alginate

**Figure 12:**
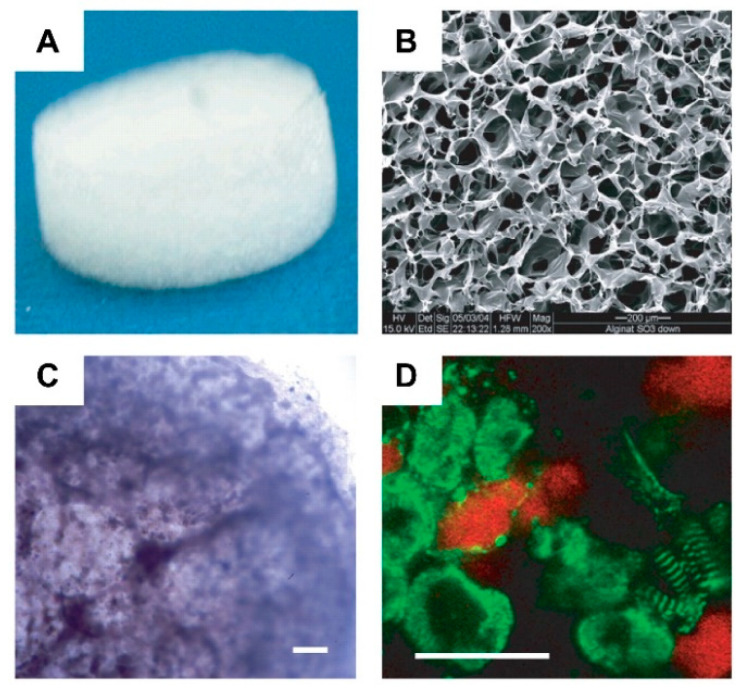
Alginate/alginate‒sulfate scaffold for cardiac patch construction. (**A**) Scaffold properties before cell seeding. (**B**) SEM image of the scaffold. (**C**) Distribution of cells in the matrix. (**D**) Cardiac cell organization in the matrix. Scale bar: 200 μm (**C**); 10 μm (**D**). Reprinted with permission from Ref. [150]. Copyright 2021 National Academy of Sciences.

Alginate is a naturally occurring polysaccharide extracted from brown seaweeds. It has been widely used for cardiac tissue engineering due to its versatility, ease of gelation, and adaptability as a biomaterial. In cardiac tissue engineering, alginate-base hydrogels can be considered a promising alternative for valve replacement techniques and cardiac regeneration [144]. However, when compared to previous materials, alginate contains a larger number of impurities and presents hydrophilicity, which negatively impacts cell proliferation and adhesion [145]. Therefore, to overcome these drawbacks, Hao et al. [145] introduced fullerenol nanoparticles into an alginate hydrogel, which created an injectable cell delivery vehicle with antioxidant activity. The obtained results suggested that the resulting fullerenol/alginate hydrogel could decrease the ROS level at the MI zone and improve the survival and retention of implanted cells, inducing angiogenesis and promoting recovery of cardiac function. In another study [146], new production methods for alginate/ECM hybrid hydrogels were developed. Here, a high G Block/ECM hybrid hydrogel with mechanical properties and no cytotoxicity was identified. This showed that alginate and ECM particles in combination could be useful in heart failure (HF) treatment and drug delivery. Injectable alginate hydrogel is also commonly used for replacing damaged ECM [147]. Through early studies that confirmed the potential of alginate-based hydrogels as ECM substitutes for cell survival promotion in acute MI [148], newer alginate-based hydrogels were designed to further mimic the cues of the heart’s ECM, where it was observed that alginate can provide tissue support, facilitating myocardial repair and function [149], as well as aiding in myocardium regeneration after myocardium infarction (MI) (Figure 12) [150]. Recently, this evidence was solidified with the confirmation of the efficacy of the biomaterial’s injection into myocardial tissue, where increased scar thickness and physical support to the healing of the tissue was observed. In certain studies, it was shown that alginate-based biopolymer injections aid in the substitution of damaged ECM [151,152].

### 3.3. Decellularized ECM in Cardiac Tissue Engineering

In heart tissue engineering and regenerative medicine, the challenge is to regenerate damaged or diseased tissue and/or create completely new organs from functional cardiac tissue [153]. This is where decellularized ECM (dECM) processes play a contributive role. Decellularization removes genetic materials (DNA) and native cardiac cells from the ECM while keeping its biochemical and structural cues. In order to produce personalized cardiac tissue, the dECM can be repopulated with an individual’s cells, which makes it a suitable scaffold for cardiac tissue engineering applications due to its versatility and utility [154]. In cardiac tissue engineering, dECM is a promising biomaterial capable of repairing cardiovascular tissue, as it effectively obtains the complex array of proteins, proteoglycans, glycosaminoglycans (GAGs), and many other components of the cardiac native tissue [155]. dECM provides ideal cues for the repair, regeneration, and remodeling of damaged myocardium [155,156]. Currently, there are still some challenging points in cardiac dECM processes, i.e., dECM recellularization and decellularization still face obstacles, including the balance between ECM preservation and cell removal for obtaining homogeneous cell distribution and enhancing the bioactivity and prevascularization of dense ECM [157].

In the following sections, we will be addressing novel techniques and applications of dECM in natural and synthetic ECMs.

#### 3.3.1. Fabrication of dECM

**Figure 13:**
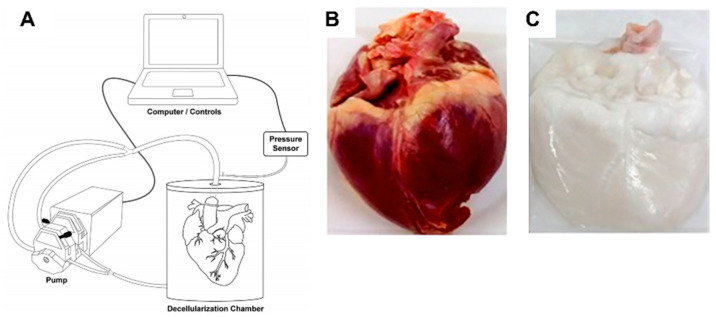
Images of decellularization chamber setup (**A**); native control porcine heart (**B**); and decellularized porcine heart (**C**). Reprinted with permission from Ref. [158]. Copyright 2017 Whioce Publishing Pte. Ltd. (Ang Mo Kio, Singapore).

To mimic the natural cell microenvironment, biomaterials such as decellularized porcine cardiac ECM (pcECM) were explored (Figure 13) [158]. Its structure, composition, and bioactivity can play a key role in cell behavior regulation. In particular, pcECM’s production served as the base material for the development of three unique scaffolds: Decellularized pcECM patch (D-Patch) [159], Novel Electrospun pcECM patch (ES-Patch) [160], and PcECM-based hydrogel (hydrogel) [161]. In all three scaffolds, both collagen and glycosaminoglycans were still major constituents and different production processes did not affect the relative quantities of the most commonly found components or collagen types in the cardiac ECM (noting that for the hydrogel collagen type VI was not found—which, according to studies conducted by Luther et al. [162], is not necessarily a negative consequence, as the knockout of collagen type VI can actually improve cardiac function and regeneration following myocardial infarction).

In a study conducted by Seif-Naraghi et al., the efficacy, safety, and biocompatibility of using an injectable matrix derived from decellularized porcine myocardium were studied [163]. Potential therapeutic effects of the matrix were assessed through a pig model, where, two weeks after MI, an injection of the matrix, saline, or no injection was given to pigs. Results showed that the group treated with the myocardial matrix had smaller fractional increases in infarct size when compared to control groups, indicating a reparative response.

#### 3.3.2. dECM and Induced Cardiac Progenitor Cells (iCPCs)

Induced pluripotent stem cells and directed differentiation techniques are common in cardiac tissue engineering, disease modeling, and regenerative therapy. In a study conducted by Ruben et al., cardiac tissue was generated and differentiated by the induction of cardiac progenitor cells in a decellularized heart scaffold [164]. It was shown that adult mouse fibroblasts can be reprogrammed to become expandable, multipotent, and induced progenitor cells (iCPCs) through the employment of forced expression, along with signaling paths such as canonical Wnt and JAK/STAT activation. This proved the safety, versatility, and functionality of iCPCs for cardiac regenerative therapy. Additionally, dECM can be enhanced by employing polymers in the cellular microenvironment, Thus, a method to dissolve the ECM into a polymer/solvent solution was suggested by A. Reid [165]. In this study, to harness the biochemical and mechanical integrity of the polymer in the ECM, the ECM, including bovine aorta and myocardium, was dissolved into a polymer/solvent solution and electrospun into a fibrous sheet. Moreover, the scaffolds were seeded with human umbilical vein endothelial cells (HUVECS) and it was found that the inclusion of aorta ECM increased the wettability of the scaffolds. This led to increased HUVEC adherence and proliferation. Lastly, with the addition of ECM, gene expression and mechanical changes were observed, demonstrating the potential of electrospun ECM/polymer bioscaffolds and their respective use in cardiac tissue engineering.

### 3.4. Synthetic Materials for ECM in Cardiac Tissue Engineering

Synthetic ECMs overcome the limitations observed in naturally derived ECMs, such as the lack of functional cardiomyocyte maturation and typical structural organization of the adult myocardium. The recreation of an artificial microenvironment similar to the native tissue can be obtained through the use of synthetic materials and techniques such as polymers, scaffolds, hydrogels, and 3D printing. In this section, we will be exploring novel synthetic ECM techniques and applications.

#### 3.4.1. PEG (Poly(ethylene glycol))

**Figure 14:**
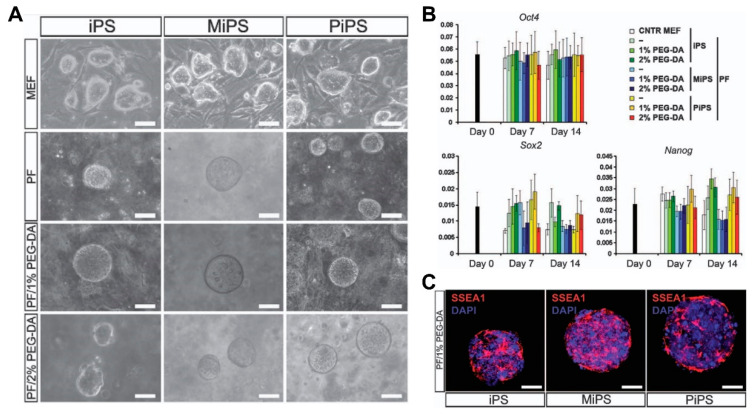
Culturing iPS cells on PEG‒fibrinogen scaffolds. (**A**) Cellular morphology of colonies based on scaffold type. (**B**) Gene expression of the cells dependent on proliferation status per scaffold. (**C**) Immunofluorescence assays of cell colonies on supplemented PEG‒DA scaffold. Scale bar: 100 μm. Reprinted with permission from Ref. [172]. Copyright 2014 Springer Nature Limited.

Due to its non-immunogenicity, PEG (Poly(ethylene glycol)) is one of the most utilized synthetic polymers for scaffold fabrication in cardiac tissue engineering. PEG is made by diacrylate-modified PEG polymerization and provides a highly modifiable platform that can be utilized to create biological functions in cardiac tissue, due to its branched structure, varied polymerization chemistry, and easy-to-modify functional groups. There were studies dedicated to the observation of cardiomyocyte‒matrix interactions in 3D environments with the use of PEG hydrogels. In a study, the viability of cardiomyocytes increased to a great extent after Arg-Gly-Asp (RGD) peptide modification [166]. In another study, a series of PEG hydrogels with various crosslinking densities were constructed and the results revealed that softer hydrogels induced ESCs’ differentiation, which showed cardiac-like functions [167]. However, such cardiac-like functions can be limited by the lack of structural organizations and electrical conductivity. In order to address these limitations, Smith et al. [168] developed scalable, graphene-functionalized topographies that possess anisotropic electrical conductivity for cardiac tissue constructs, engineering its functional and structural composition at a macroscopic level. Moreover, PEG-based hydrogels were employed with other hydrogels or nanoparticles for the creation of hybrid polymer blends that can better replicate the ECM of cardiac tissue [169,170] or be utilized for the creation of 3D cardiac tissue composed of cellular-derived CMs for the support of engineered cardiac tissue and vasculature [171]. Recently, hybrid PEG scaffolds were created and seeded with therapeutic cells under optimal stiffness conditions for cell survival and tissue regeneration (Figure 14) [172].

#### 3.4.2. Poly(Lactic Acid)—PLLA Scaffolds

**Figure 15:**
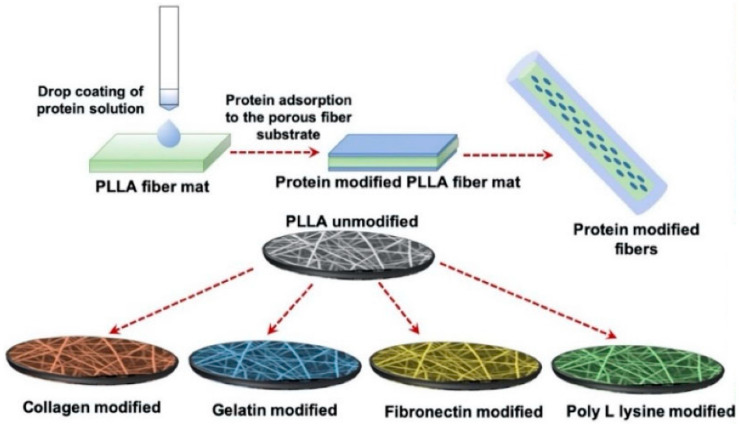
Schematic of protein functionalization on porous poly(lactic acid)—(PLLA) scaffolds. Drop coating of ECM proteins for surface functionalization in PLLA scaffolds. Reprinted with permission from Ref. [162]. Copyright 1996–2021 MDPI.

Polylactide (PLA) is a hydrolyzed aliphatic semicrystalline polyester and polymerized by lactic acid that presents a variety of uses in tissue engineering due to its biodegradability [136]. In particular, one of its four different stereoisomeric forms, poly(L-lactic acid), is more commonly used in cardiac tissue engineering. As briefly mentioned, PLLA is known for its nontoxic, biocompatible, biodegradable capabilities and fitting mechanical properties [173]. Due to its slow degradation, though, PLLA faces certain limitations in cardiac tissue engineering [174]; however, certain studies grew cells in PLLA scaffolds that were able to degrade without further help from enzymes or catalysts, when inserted in vivo [175]. In other studies, porous nanofibrous PLLA scaffolds were developed to engineer cardiac constructs with CPCs derived from mouse embryonic stem cells (ESCs) and the results revealed improved cell attachment, differentiation, and extension [176]. Recently, electrospun porous PLLA scaffolds were developed and modified with various ECM-derived proteins, with adult human cardiac fibroblasts (AHCF) on modified surfaces. The results showed that, regardless of surface modifications, great cell adhesion and proliferation of the cells were observed on the porous PLLA fibers (Figure 15) [162]. Moreover, PLLA scaffolds can also be applied with other hydrogels and scaffolds, such as polycaprolactone (PCL), for the development of superior hybrid scaffolds in cardiac tissue engineering [177].

#### 3.4.3. Poly(2-hydroxyethyl methacrylate)—PHEMA

**Figure 16:**
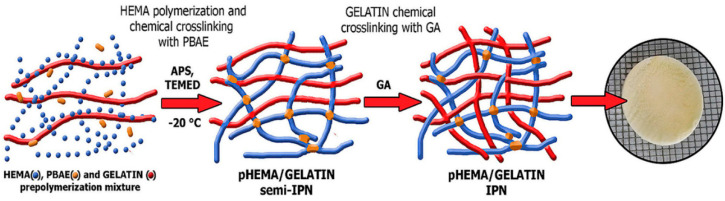
The construction of a novel biodegradable PHEMA/gelatin hybrid scaffold for tissue regeneration. Reprinted with permission from Ref. [183]. Copyright 2021 Elsevier B.V.

Poly(2-hydroxyethyl methacrylate) (PHEMA), a hydrophilic polymer composed of pendant hydroxyl groups, has been used in cardiac tissue engineering. PHEMA hydrogels are generally considered biocompatible; however, certain studies raised the question of whether such constructs can be biocompatible over a long period of time [178,179]. Additionally, PHEMA-based hydrogels or composites were tested as alternatives for natural scaffolding materials in engineered cardiac tissue with the use of hPSC-CMs, in which the survival and increased proliferation of the respective cells in cardiac tissue was enhanced; however, maturation of hPSC-CMs was not observed [180]. Recently, various PHEMA-derived hydrogels and scaffolds were studied, with a special focus on cellular differentiation and maturation. In a study conducted by Lao et al. [181], two types of PHEMA stents were studied for the differentiation of adipose-derived stem cells (ASCs) into myocardial cells. Based on the different surface and cross-sectional morphological characteristics of the studied groups, it was shown that a PHEMA stent structure, with low water content and a high number of matrixes, induced an increased level of ASC differentiation in myocardial cells. PHEMA can also be frequently copolymerized with other materials [182], such as poly(methyl methacrylate) (MAA), for more specialized studies in cardiac tissue engineering. Moreover, recent studies also focus on the combination of PHEMA hydrogels with other hydrogels of weaker mechanical properties, such as gelatin hydrogels, in order to form novel scaffolds with improved mechanical properties, biocompatibility, and degradation rates (Figure 16) [183].

## 4. Geometry

Heart tissue is a highly organized structure consisting of cardiomyocytes aligned unidirectionally. These cardiomyocytes form cardiac fibers that produce and propagate anisotropic action potentials and contractions in parallel based on the muscle fiber alignment. Culturing cardiomyocytes in natural or synthetic EC without geometrical cues could not make structured matured cardiac tissue due to the random distribution of cardiomyocytes in the matrix since the cardiomyocytes in the engineered tissue are not organized according to the native tissue. Microfabrication technologies such as microcontact printing and microtopology were developed to align cells to form matured cardiac tissue. Incorporating patterns and topological confinements on two-dimensional substrates and three-dimensional scaffolds is one way to provide geometrical cues for the fabrication of mature cardiac tissue. Patterning bioactive molecules on the surface was challenged by microcontact printing and topological guidance for providing geometrical guidance was also challenged by microfabrication technologies based on the photolithography. In addition, providing strain on cardiac tissue can serve as another physical cue that provides a cellular milieu similar to the native myocardial environment. In the following section, we will discuss representative engineering techniques that provide geometrical cues based on the various scales in assembling engineered cardiac tissue.

### 4.1. Patterning Geometry in Nanoscale

**Figure 17:**
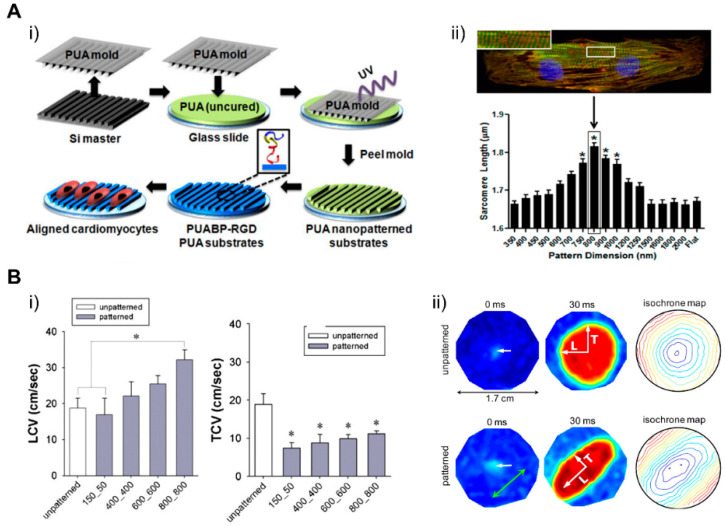
(**A**) Scheme for fabrication of RGD-functionalized nanopatterned cell culture array (**i**). The sarcomere length of cardiomyocytes on the nanopattern (* *p* < 0.05 ) (**ii**). (**B**) Longitudinal and transverse velocities of the action potential on nanopatterned surface (* *p* < 0.05 ) (**i**). Anisotropic action potential propagation (**ii**) within the patterned tissue compared to the control (**ii**). Reprinted with permission from [17,18]. Copyright 2016 American Chemical Society and Copyright 2021 National Academy of Sciences, respectively.

Cardiac ECM geometry patterned at a nanoscale can modulate engineered cardiac tissue function and structure [184], in which nanotopological features of cardiac ECM can have an influence on the function of the cardiac tissue, which is supported by recent studies where cardiomyocytes were cultured on submicrometer patterns [185]. In a study by Kim et al., a scalable, nanotopographically controlled model of the cardiac matrix that mimics in vivo ventricular organization was constructed. Results showed that, when guided by an underlying hydrogel with nanoscale mechanical cues, the tissue constructs displayed contractility and anisotropic action potential similar to that of the native cardiac tissue [18]. The same was observed in a study by Carson et al., where hiPSC-CMs cultured on varying widths of nano ridge/groove surfaces, and functionalized with RGD peptides, showed CM alignment and a mature CM-like morphology [17]. When it comes to the creation of various cardiac fiber widths, similar to the native cardiac tissue, electrospinning techniques were utilized to form nanofibers that have the potential to further induce cellular fiber alignment and contractile function optimization [184,186,187,188]. Confined attachments also seemed to induce alignment in cells and lead to better structural and functional coordination between cells, improving the overall function of engineered tissue (Figure 17) [17,18]. Moreover, nanopatterned geometrical confinement can be utilized for the study of cardiac development, drug-induced developmental toxicity, and embryonic spatial patterning. Such potential was studied by Ma et al., in whose study, PEG-patterned substrates were used to geometrically confine hiPSCs and induce mechanical stress. It was shown that biophysical and biochemical cues both were able to induce the beating of the cardiac microchamber with self-organizing lineage specification [189]. Regarding cardiac development, the clustering behavior of cell-surface receptors has been commonly studied through nanoscale approaches. In a study conducted by Hawkes et al., the role of integrin clustering was studied during cardiomyocyte maturation and adhesion with the usage of focused ion beam and electron-beam lithography nanopatterning [190]. Recently, researchers have focused on the development of efficient electrical propagation in cardiac tissue through the development and research of nano constructs, materials, and nano based polymeric devices that are able to support cell differentiation, alignment, and proliferation [191,192].

### 4.2. Patterning Geometry in Microscale

**Figure 18:**
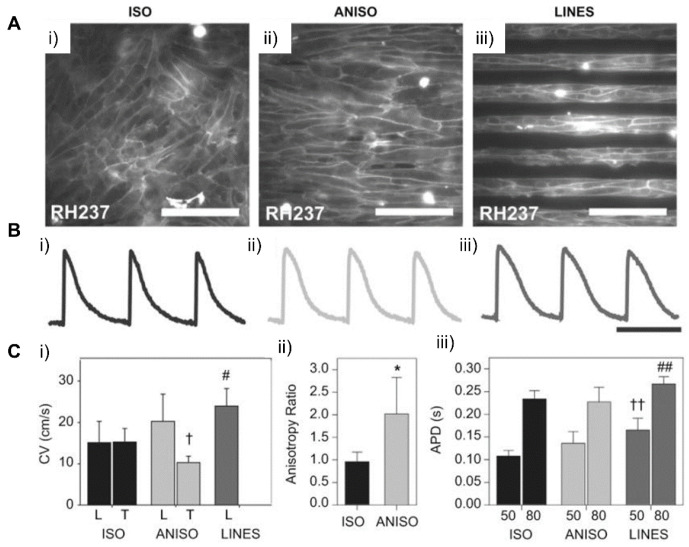
Engineered cardiac tissue generated on different micropatterned surfaces and the functionalities of the tissue. (**A**) Seeded rat ventricular cardiomyocytes in control (**i**), confluent anisotropic pattern (**ii**), and 20 μm spaced pattern (**iii**), respectively. (**B**) The beating signals of cardiomyocytes corresponding to each pattern in (**A**). (**C**) Contraction velocities (**i**), action potential propagation (**ii**), and action potential duration (**iii**) of cardiomyocytes in each pattern. Statistic significant is based on *p* < 0.05 versus ISO for *, † and #; versus ISO and ANISO for ††; and versus ANISO for ##. Reprinted with permission from Ref. [193]. Copyright 2012 Elsevier Ltd.

Patterning on the two-dimensional surface of substrates, where cells are cultured to engineer cardiac tissue, is one of the most popular ways to incorporate geometrical cues in the cellular environment. Microscale patterns, especially, are known to regulate the structural alignment and maturation of cells. This possibly arises from the fact that patterns at this scale confine the boundary of cells affecting cytoskeleton arrangement, which affects the nuclear shape and subsequent phenotype changes [191]. In a study by Max et al., rectangular patterns with various widths and aspect ratio caused alignment of the human embryonic stem cell-derived cardiomyocytes (hESC-CMs) along with the widths in the range of 30 to 80 μm. This showed that the microgeometry that confines cell adhesion to the surface affects the arrangement of cells and intracellular structures (Figure 18) [193]. In another study, NRVMs cultured on various micropatterned fibronectin aligned in varying anisotropic degrees exhibited more natural CM-like Ca^2+^ transient features, such as a decreased Ca^2+^ base line during diastole and increased Ca^2+^ influx for each cardiac cycle, indicating an improvement in excitation-contraction coupling (ECC) [194]. These results demonstrated that micropatterns within extracellular matrices that confine boundaries of cells and cellular attachments can affect the muscle tissue structures on cellular and subcellular levels and improve its functions, such as force generation or excitation‒contraction coupling. 3D micro constructs at a defined geometry were fabricated for the repair and regeneration of cardiac tissue [195,196]. In a study conducted by Gaetani et al., a micro construct model containing cardiac-derived myocyte progenitor cells (hCMPCs), modified ECM, with RGF peptide-modified sodium alginate, was developed. The results showed the promotion of in vitro hCMPCs into cardiomyocyte-like cells’ differentiation and their migration out of the micro construct [195]. Considering that the majority of inter cellular interactions occur at a microscale, current research has focused on the development of scaffolds and micro constructs that can better guide cell behaviors. For instance, soft lithography has been utilized to originate microscale molds that can be utilized to pattern hydrogels or materials such as PEG, PLLA, etc. [197,198]. For the engineering of functional cardiac tissue, as mentioned, directing cell fate is crucial. Hence, the usage of biomaterials alongside micropatterning and micro construct development for the recapitulation of the complex native cardiac tissue structure [199,200].

### 4.3. Patterning Geometry in Multiscale

**Figure 19:**
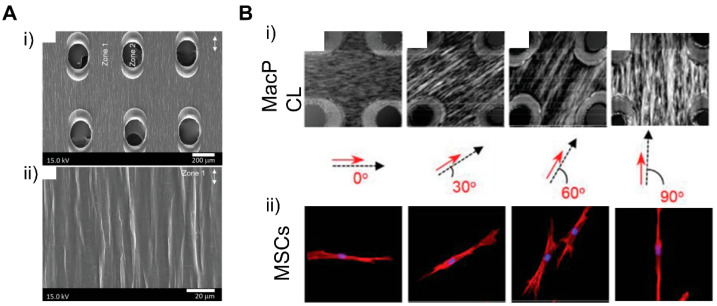
The engineered dual-structured scaffolds mimicking native myocardium. (**A**) Close view of macrohole array (**i**) and microwrinkles on the surface (**ii**). (**B**) Polymer sheets with aligned macroholes and rotating microwrinkles on the surfaces by degrees (**i**). The MSCs seeded on each layer aligned in the direction of the microwrinkles (**ii**). Reprinted with permission from Ref. [202]. Copyright 2019 WILEY-VCH Verlag GmbH & Co. KGaA.

Models of the natural myocardium environment but with a greater extent can be achieved by integrating different scales of geometries and topographical cues. Luna et al. presented a winkled culture platform that mimics the anisotropic and multiscale architecture of the heart [201]. The culture platform that molded metal wrinkles using PDMS had a wrinkle thickness of 20 nm to 10 μm and an average wrinkle thickness of 800 nm to 1 μm. As a result of applying multiscale biomimetic topographic cues to cardiomyocytes, it was confirmed that multiscale patterns induced the cellular and subcellular alignment of cardiomyocytes. In another study, Abadi et al. incorporated submicrometer topographical features of mature cardiomyocytes into a microscale structure by imprinting primary human CMs cultured on cylindrical micropatterns [185]. It was shown that the submicrotopographical features of mature cardiomyocytes enhanced the differentiation of iPSCs into iPSC-CMs. Furthermore, the CMs exhibited more mature phenotypes such as the expression of maturation markers, beating properties, and mitochondrial distribution. In addition to the nano/micro multiscale patterns, Zhang et al. applied micro/macro multiscale patterns to fabricate cardiac tissue. They modeled the changing direction of cell alignment in myocardium across the heart wall thickness by stacking dual-structured layers with macrohole arrays and microarray patterns [202]. The macroholes improved cell alignment compared to micropatterned surfaces without holes and enabled cell distribution across the structure when hMSCs were seeded. The cells aligned in the direction of the micropattern model the complex tissue structure of natural myocardium, in which the orientation of cell alignments changes along with the depth of the myocardium wall (Figure 19). These studies showed that replicating the natural myocardial environment in which cells exist can lead to further improvement of the morphological features of cardiac tissue.

### 4.4. Dynamic Patterning

**Figure 20:**
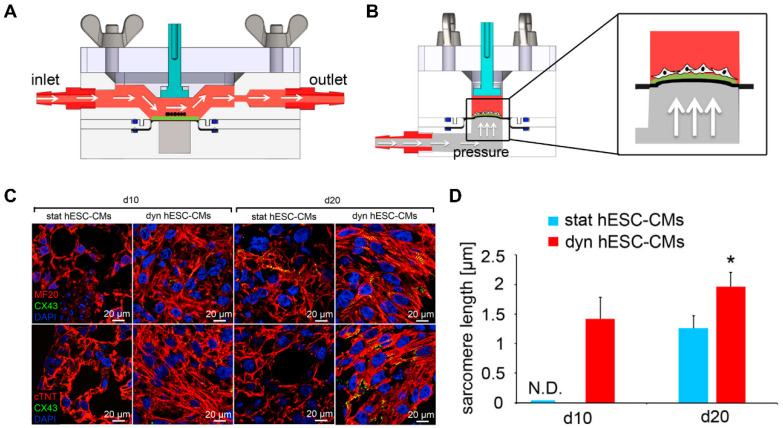
Flow chamber of bioreactor system in a cross-sectional view (**A**) and mechanism of vacuum-driven physiological stretch profile (**B**). Immunofluorescence images of cardiomyocytes in a static or dynamic system (**C**). Quantification of sarcomere length in D10 and D20 of static or dynamic groups (**D**). N.D. means not available. * *p* < 0.01 versus stat hESC-CMs at the same time point. Reprinted with permission from Ref. [207]. Copyright 2017 Elsevier B.V.

In addition to simply providing the microstructure of native tissue by patterning, microphysiological devices were developed to actively simulate the physical environment of a specific microenvironment of cells [203]. In particular, forces present in the heart, such as flow and tensile strength, can affect aspects of cell physiology, such as the alignment and maturation of cardiomyocytes. Kolanowski et al. developed a microfluidic system that induced cyclic pulsatile hemodynamic forces to induce cardiac maturation [204]. It was shown that hemodynamic forces induced cell alignment and increased contractility. In addition to the iPSC-CMs becoming rod-like in shape, morphological maturation markers such as an increase in cell size and sarcomere length were indicated. Since the native myocardium has a tensile force along with hemodynamic forces [205], a microphysiological platform was developed that can give these two forces. Lux et al. fabricated a microphysiological system using periodic stretch and perfusion stimulation on a 3D heart patch [206]. It was shown that the stimulation of cyclic stretch induced CM alignment along the stretch axis and improved the contractile function and gene expression of cardiomyocyte markers. Shen et al. developed a bioreactor system that uses pulsatile flow and cyclic strain to mimic the microenvironment of the heart (Figure 20) [207]. Combination of 1.48 mL/min pulsatile flow with 5% cyclic strain synergistically improved the alignment and maturation of cardiomyocytes. On the other hand, biologically inspired systems, i.e., organ-on-a-chip (OOC) that mimic human physiology in biological stand points, were also developed. The merit of the OOC system arises from the integrity of multiple organs with fluidic environment that have key characteristics of a native system. For instance, Chramiec et al. showed that the OOC model integrated with bone tumor and cardiac tissue was an efficient platform to evaluate not only the effect of anti-tumor drugs but also its toxicity on cardiac tissue. This work suggested that OOC systems could be a valuable platform to screen novel drug candidates in pre-clinical models. These studies suggested that microphysiological devices that stimulate physical forces, including hemodynamics and tensile forces, could regulate functional maturation such as contraction and gene expression, as well as structural maturation such as cell alignment and morphology.

### 4.5. Patterning 3D in Macroscale

Creating a macroscale or whole heart is the ultimate challenge when fabricating cardiac tissue. Scaling up from nano or microscale to macro is not a solution for creating a macroscale heart. For fabricating a heart in vitro, 3D printing technologies will be the baseline technologies. Advances in 3D printing technology made it possible to engineer cardiac tissue from the microscale to the macroscale [13]. In particular, this technology provides insight for the scaling-up of the constructs including microphysiological devices, patterned tissues, and implantable scaffolds [208,209]. For the macroscale fabrication, at least three factors were also considered: coculture of heart cells, assembly of organ specific ECMs, and fabrication of heart geometry.

**Figure 21:**
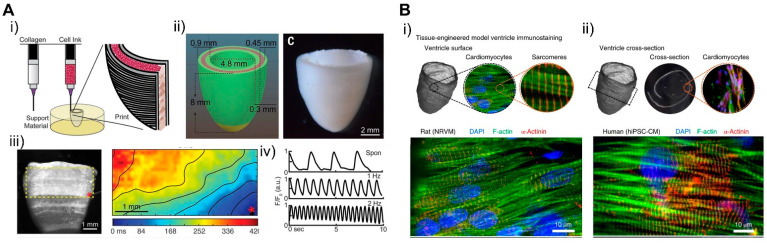
(**A**) Fabrication of cell-loaded 3D human ventricle model using 3D bioprint (**i**). Computer-aided design (CAD) of ventricle with area to be laden with cells marked in pink and the generated ventricle tissue according to the design (**ii**). Calcium mapping of the subregion (yellow window) in (**iii**) exhibits directional propagation of calcium wave with conduction velocity of 1.97 cm/s. Red asterisk (*) indicates the start point of propagation. Beating rate of the 3D chamber, (**iv**) without electrical pacing (**top**) and with electrical pacing of 1 and 2 Hz (**middle and bottom**), respectively. (**B**) The surface (**i**) and cross-sectional (**ii**) view of iPSC-CMs aligned within a three-dimensional nanofiber chamber. Reprinted with permission from Refs. [212,213]. Copyright 2019 American Association for the Advancement of Science and Copyright 2018 Springer Nature Limited, respectively.

For the cells, Maiullari et al. fabricated 3D bioprinted cardiac tissue with a heterogeneous multicellular structure consisting of human umbilical vein endothelial cells (HUVEC) and iPSC-CMs [171]. Cells were encapsulated in hydrogel containing alginate and PEG-fibrinogen (PF), and a microfluidic printing head can be used to precisely tune the 3D spatial deposition to ensure high print fidelity and resolution. In 3D bioprinted tissue, HUVEC developed an endothelial-like structure and iPSC-CM showed unidirectionality compared to the bulk group. For ECMs, various types of bioinks recapitulating hearts developed with different composition and stiffness. In a study by Shin et al., dECM and PEG composed hydrogel bioinks were developed and cured for optimized mechanical stiffness showcasing shape fidelity, higher cell viability, and adaptability to various printing conditions, which enabled the construction of fibrotic cardiac tissue [210]. Moreover, cardiac ECM (cECM) containing anisotropic silk-based scaffolds were developed with tunable architectures and mechanical properties, which demonstrated the promotion of a functional and physiologically relevant cardiac tissue phenotype [211]. To fabricate a structurally similar tissue using 3D bioprinting technology, Lee et al. developed a pH-based gelation bioink to generate a 3D-printed microfibrous collagen tissue structure (Figure 21A) [212]. The scaffold can be designed in a patient-specific way using microcomputed tomography and displays synchronized contraction and considerable wall thickening up to 14% during systole. In particular, freeform reversible embedding of suspended hydrogels (FRESH) can be used to simulate human hearts at various scales from capillaries to large organs.

Controlling the geometry of the fabricated engineered cardiac tissue is also important. A study by MacQueen et al. generated a chambered structure using nanofibers that provided topographical guidance cues to cell assembly (Figure 21B) [213]. The resulting chambered structure exhibited anisotropic tissue alignment, as seen in the micropatterned monolayer, and produced chamber-level contractile function corresponding to 1/50‒1/250 fold of rodent and human ventricle ejection fraction. In another study, iPSCs were printed together with natural ECM proteins in order to overcome the low proliferating rate of cardiomyocytes and to achieve continuous tissue within a 3D scaffold [214]. The iPSCs were cultured until they reached sufficient cell density, then differentiated into cardiomyocytes showing continuous tissue structure and action potential propagation. The tissue architecture expressed chamber-level beating reconstructing pressure and volume relationship in response to drugs. Such three-dimensional structures show chamber-level beating and ejection fractions reconstructing the pressure‒volume relationship in vitro, which suggests them as good candidates for drug screening and for investigating the physiology of cardiac tissue and replacing animal models and clinical trials. This study showed that 3D bioprinting can simulate complex structures such as the heart from macroscale to microscale and can be used in the field of cardiac tissue engineering.

## 5. Computational Cardiology

In the previous section, we discussed how the heart can be modeled in vitro by a combination of cells, ECM, and geometry. However, due to the complexity of the human heart, success has not yet been achieved. Thus, computer modeling could be an alternative way to compensate for the gap between current experimental results and the real heart physiology.

Modeling of the heart in silico showed the possibility of unveiling concealed properties of a cardiac system in clinical and in vitro tests. Since the first presentation of a computational model for (cardio)myocytes in 1962 by Noble, in silico modeling of heart has advanced our knowledge of the heart more than any other organ system [215,216]. Computational modeling of cardiac cells can provide a better understanding of experimental and clinical data. Computer modeling also allows us to link the molecular and genetic mechanisms of disease to their pathological outcome. Combined with advancements in medical imaging, computer simulation starts to incorporate the specific geometry of an individual heart into modeling, providing a more accurate diagnosis of a patient’s heart. This also enables us to optimize the surgery plan by minimizing risk and side effects.

### 5.1. Cardiac Cellular Model

**Figure 22:**
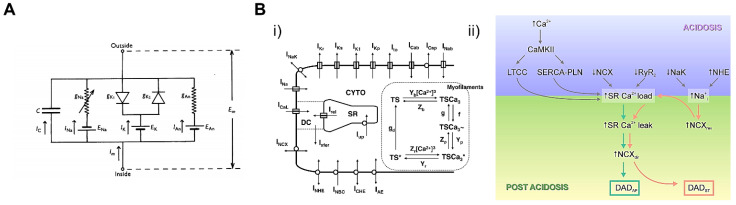
Schematic of computational cellular models. (**A**) The Purkinje cell model is modified from the Hodgkin‒Huxley model. (**B**) A human cardiomyocyte model to represent action potential, calcium handling, force generation, and intracellular pH (pH_i_) regulation by employing the mathematical formula of ion pumps and exchangers (**i**). Schematic representation of the simulated interaction of cellular components in acidosis and post-acidosis (**ii**). Reprinted with permission from [215,221]. Copyright 1962 The Physiological Society and Copyright 2013 Elsevier Ltd., respectively.

Computational cellular models refer to physically and physiologically constrained mathematical frameworks that represent phenotypic whole-cell functions, such as action potential and calcium transient, and excitation‒contraction coupling (E‒C coupling). Since the first cardiomyocyte model in 1962, which modified the Hodgkin and Huxley model of the ion current in a giant squid axon, computational cardiac models have advanced by integrating experimental and clinical data (Figure 22A) [215,216]. Recently, computational cardiomyocyte models with a wide range of physiological relevance have become available. Mathematical models of the subcellular physiological function of ion channels and enzymes are incorporated into a bigger framework of whole-cell function such as manifested action potential and calcium transient [217,218,219]. Heijman et al. presented computational models integrating action potential and calcium homeostasis. It was shown that the stimulation of β-adrenergic pathways was closely related to different β receptor isoforms and Ca^2+^/Calmodulin kinase (CaMKII) interaction [220]. Lascano et al. constructed a detailed cardiomyocyte model to simulate the postacidotic effect on the action potential by simulating the excitation‒contraction (E‒C) coupling process (Figure 22B) [221]. These studies revealed the molecular mechanism of change in whole-cell electrophysiology during biophysical events like βARS and acidosis. By simulating the functions of subcellular components, electrophysiological and electromechanical models can be used to estimate the effects of drugs and gene mutations on cardiomyocytes.

Cardiotoxicity is one of the critical features of drug development such as anticancer or anti-arrhythmogenic drugs. Computational simulation plays a key tool in screening novel drugs and evaluating their effect on the heart. There is a functional change in cardiomyocytes during the development of cardiac diseases [222]. For example, the effects of drugs on single or multiple channels can be predicted by incorporating experimental data into virtual cardiomyocyte models. Fernandez-Chas et al. presented a ventricular cardiomyocyte model to estimate the mechanism of cardiotoxicity of anticancer drug doxorubicin (DOX) and its metabolite doxorubicinol (DOXL). The effect of DOX and DOXL on the individual ion channel on action potential duration (APD) and Ca^2+^ dynamics was evaluated using a ventricular excitation‒contraction (E‒C) coupling model [223]. The therapeutic window of a drug could also be predicted by simulating the action potential of ventricular myocytes. The kinetics of cardiac sodium channels caused by anti-arrhythmic drugs could predict the action potential of ventricular myocytes [224].

### 5.2. Whole-Heart Model

Whole-heart modeling and simulations significantly improved our understanding of heart function. Recent advances in computational models and simulation allow for interpreting a myriad of experimental data and correlating them to important cardiac mechanisms [217]. Incorporation of a valid mathematical model of cardiomyocytes into a multiscale level led to further progress in whole-heart modeling. In addition, high-resolution medical imaging of patients’ hearts also contributed to developing more precise modeling of the whole heart.

The development of whole-heart models involves the conversion of the anatomical structure of the heart into a digitized virtual structure where the calculation of the physical properties can be conducted [225,226]. In the early whole-heart models, the heart geometry was constructed based on histological sectioning, interpolating the gap between histological slide sections [227]. The advance of high-resolution medical imaging methods such as computed tomography (CT) and magnetic resonance images (MRIs) facilitated detailed and user-specific heart geometry models [228,229]. Here, image-based heart geometry is converted into small unit volumes with geometrical shapes, i.e., meshes, allowing us to compute the differential equations of electric propagation or mechanical contraction. To solve such equations on a whole-heart geometry, the equations are discretized by small unit volume and computational solutions are combined to represent whole organ action potential and mechanical movement. Since physical properties (electrical, mechanical, and fluidic properties) have different requirements in terms of the degree of discretization (i.e., element size) and element types, different mesh structures are required [230]. Information on fiber orientation is also required to simulate the direction of electric propagation and mechanical contraction along the laminar structure of the heart. Digitized information on fiber orientation was also obtained from histological sectioning or DTMR images [231].

The hemodynamics of the heart is an important index to monitor heart function and drug effects. Implementing hemodynamic models in vitro involves many technical challenges, so computational models were developed to predict the hemodynamics of the heart. Hemodynamic models predict blood flow within the heart under a repeated expansion‒contraction cycle, coupled with cardiac electromechanics [226,232]. In particular, computational fluid dynamics (CFD) models based on the Navier‒Stokes equation were used to simulate the pumping of the heart [233]. Generally, image-based kinematic models were used as an input for blood flow simulation by imaging the motion of the endocardium. This model is suitable for simulating static conditions such as a normal heart, but has limitations in terms of the simulation and prediction of dynamic conditions such as afterload [234]. Mechanofluidic models developed by adding the mechanics of the heart to image-based models can dynamically simulate altered states and provide more accurate information on overall characteristics such as flow patterns, blood retention times, and flow stagnation than image-based kinematic models. For example, Augustin et al. developed a mechanofluidic model based on patient-derived data such as MRI, ECG, and invasive pressure recording [235]. This study showed that automated models can simulate the patient’s heart rate and accurately reproduce the clinical data. Another mechanofluidic model based on MR images utilized the deformation of canine left ventricles obtained from electromechanical models as an input for hemodynamic modeling. The model successfully reproduced the difference between normal and diseased canine hearts that was observed in in vivo experiments [228]. These studies suggested that modeling of the electrophysiology, mechanical properties, and hemodynamics of the heart can be used to predict the assembly of engineered cardiac tissues and their maturation.

## 6. Conclusions

This review recapitulates recent progress on cardiac tissue engineering by indexing the preparation techniques of matured cardiac tissue, from simple to complex. In the section on cardiomyocytes, we described how to prepare intact cardiomyocytes from other cells that are commonly available with no limitation. For instance, cardiomyocytes from other mammals were isolated to study cardiac physiology and then animal ESCs were also used to differentiate them into cardiomyocytes. Such efforts continued the use of human ESCs to establish human cardiomyocytes. More recently, the development of iPSCs and their use in cardiac engineering opens up a new avenue to conduct experiments for cardiac engineering.

The establishment of human cardiomyocytes is the first step in constructing fully functional heart tissue, which has led researchers to develop a number of methods for matured cardiomyocytes. Biochemical and gene editing technologies were utilized in mature cardiomyocytes, resulting in meaningful success as well as unsatisfied fidelity. Such outcomes made us focus more on other components of the heart system, including cardiac ECM, geometry, and mechanical force. The ECM is not just a structural scaffold of cardiac cells, but plays a critical role in managing their homeostasis. This led us to investigate natural and synthetic biomaterials to develop the enhanced ECM that facilitates cardiac tissue maturation.

In addition to biomaterials, geometric and mechanical environments of cardiac systems were studied, which allowed us to improve the maturation of cardiac tissue. Here, we discussed some examples of using such factors from the protein to the organ level in cardiac tissue engineering. Recent progress on computer modeling initiated a new tool to study cardiac physiology that is very complex at multiscale levels. The use of computer simulation allowed us to explore many virtual experiments that are not easily tested, mainly due to the experimental limitations of the current method. Our review provides an understanding of multiscale technology, which could be highly beneficial to move cardiac tissue engineering forward. Our review only cites a few studies related to methods of cardiac tissue generation; we would be pleased to encounter other review papers that would help increase our understanding of the current technology and give new perspectives on cardiac tissue engineering.

## Figures and Tables

**Table 1:** Advantages and disadvantages of cardiomyocyte cell source.

Cell Source (Type)	Advantages	Disadvantages
Adult rat ventricular myocyte (ARVM)	Easy to obtain Mature	Xenogenic
Neonatal rat ventricular myocyte (NRVM)	Easy to obtain	Xenogenic Immature
Adult porcine ventricular myocyte	Genetically closer to human cells than rodent	Xenogenic
Human embryonic stem cell-derived cardiomyocyte (hESC-CM)	Same species Unlimited cell source	Ethical issue Allogenic Immature
Human-induced pluripotent stem cell-derived cardiomyocyte (hiPSC-CM)	Same species Unlimited cell source Autologous	Immature

**Table 2:** Advantages and disadvantages of natural and synthetic materials (ECM) in cardiac tissue engineering.

	Name	Advantages	Disadvantages
**Natural** **materials**	Collagen; decellularized ECM	Biodegradable and biocompatible	Slow gelation, weak strength
	Fibrin gel	Biodegradable and biocompatible	Fast degradation in vivo and slow gelation
	Matrigel	Similar to native ECM structure	Potentially carcinogenic
	Chitosan	Structurally similar to heart tissue components	Insufficient support for cardiac tissue growth
	Alginate	Biodegradable, biocompatible, non-toxic, and cost effective	Weak cell adhesion and proliferation
**Synthetic materials**	PHEMA	Biocompatible	Non-degradable and mismatch of modulus
	PEG	Biocompatible, bioinert, and FDA approved	Non-degradable, low cell adhesion, and toxicity
	Polyamides	Versatility in chemical modification, fast gelation time, and injectability	Non elastic and non-degradable

## Data Availability

Not applicable.
